# RETRACTION: Sesamin Enhances Nrf2‐Mediated Protective Defense against Oxidative Stress and Inflammation in Colitis via AKT and ERK Activation

**DOI:** 10.1155/omcl/9830464

**Published:** 2026-01-05

**Authors:** Oxidative Medicine and Cellular Longevity

RETRACTION: X. Bai, X. Gou, P. Cai, C. Xu, L. Cao, Z. Zhao, M. Huang, and J. Jin, “Sesamin Enhances Nrf2‐Mediated Protective Defense against Oxidative Stress and Inflammation in Colitis via AKT and ERK Activation,” Oxidative Medicine and Cellular Longevity 2019, no. 1 (2019): 2432416, https://doi.org/10.1155/2019/2432416.

The above article, published online on 26 August 2019 in Wiley Online Library (wileyonlinelibrary.com), has been retracted by agreement between the authors, the journal Editor‐in‐Chief, Jeannette Vasquez‐Vivar, and John Wiley & Sons Ltd. The retraction has been agreed due to concerns identified by the authors with the figures, also raised on PubPeer [1]. Specifically:

• The SSM (2 h) GR blots in Figure 4a appear identical to the SSM (8 h) GAPDH blots shown in Figure 5a when flipped horizontally.
• In Figure 5d, the HO‐1 blots appear identical to the GCLM blots.

After requesting further clarification regarding the errors, the authors requested the retraction of the article due to a lack of confidence in the reliability of the results. After assessment by the Chief Editor, the article is retracted.

The authors agree to the retraction.
